# Pancancer analysis of a potential gene mutation model in the prediction of immunotherapy outcomes

**DOI:** 10.3389/fgene.2022.917118

**Published:** 2022-08-26

**Authors:** Lishan Yu, Caifeng Gong

**Affiliations:** ^1^ Yanqi Lake Beijing Institute Mathematical Sciences and Applications, Beijing, China; ^2^ Yau Mathematical Sciences Center, Tsinghua University, Beijing, China; ^3^ Department of Medical Oncology, National Cancer Center/National Clinical Research Center for Cancer/Cancer Hospital, Chinese Academy of Medical Sciences and Peking Union Medical College, Beijing, China

**Keywords:** immune checkpoint blockade, immunotherapy, gene mutation signature, statistical modeling, biomarker

## Abstract

**Background:** Immune checkpoint blockade (ICB) represents a promising treatment for cancer, but predictive biomarkers are needed. We aimed to develop a cost-effective signature to predict immunotherapy benefits across cancers.

**Methods:** We proposed a study framework to construct the signature. Specifically, we built a multivariate Cox proportional hazards regression model with LASSO using 80% of an ICB-treated cohort (*n* = 1661) from MSKCC. The desired signature named SIGP was the risk score of the model and was validated in the remaining 20% of patients and an external ICB-treated cohort (*n* = 249) from DFCI.

**Results:** SIGP was based on 18 candidate genes (NOTCH3, CREBBP, RNF43, PTPRD, FAM46C, SETD2, PTPRT, TERT, TET1, ROS1, NTRK3, PAK7, BRAF, LATS1, IL7R, VHL, TP53, and STK11), and we classified patients into SIGP high (SIGP-H), SIGP low (SIGP-L) and SIGP wild type (SIGP-WT) groups according to the SIGP score. A multicohort validation demonstrated that patients in SIGP-L had significantly longer overall survival (OS) in the context of ICB therapy than those in SIGP-WT and SIGP-H (44.00 months versus 13.00 months and 14.00 months, *p* < 0.001 in the test set). The survival of patients grouped by SIGP in non-ICB-treated cohorts was different, and SIGP-WT performed better than the other groups. In addition, SIGP-L + TMB-L (approximately 15% of patients) had similar survivals to TMB-H, and patients with both SIGP-L and TMB-H had better survival. Further analysis on tumor-infiltrating lymphocytes demonstrated that the SIGP-L group had significantly increased abundances of CD8^+^ T cells.

**Conclusion:** Our proposed model of the SIGP signature based on 18-gene mutations has good predictive value for the clinical benefit of ICB in pancancer patients. Additional patients without TMB-H were identified by SIGP as potential candidates for ICB, and the combination of both signatures showed better performance than the single signature.

## Introduction

Immune checkpoint blockade (ICB) is regarded as a major breakthrough in increasing the survival time of patients undergoing cancer treatment (Couzin-Frankel, 2013a; Couzin-Frankel, 2013b; Adachi and Tamada, 2015; Ito et al., 2015; Kelly, 2018). In reality, available immunotherapeutic agents are expensive. Only a few patients benefit from ICB treatment, and a few patients might experience adverse effects, such as hyperprogressive disease (HPD) (Corsello et al., 2013; Wang et al., 2018; Martins et al., 2019; Thapa et al., 2019; Jin et al., 2020). For the safety of patients and to avoid ineffective treatment, it is significant to identify patients who will benefit from ICB treatment (Ventola, 2017).

In recent years, an increasing number of studies have focused on predicting the response of patients to ICB. These studies developed biomarkers to predict the prognosis of patients treated with ICB (Topalian et al., 2016; Havel et al., 2019; Otoshi et al., 2019). PD-L1 expression, microsatellite instability (MSI), and tumor mutation burden (TMB) are the most investigative biomarkers for predicting the response to immunotherapy (Patel and Kurzrock, 2015; Hugo et al., 2016; Goodman et al., 2017; Chang et al., 2018; Miao et al., 2018; Samstein et al., 2019). However, the use of the three biomarkers in the clinic remains limited; thus, only a small portion of patients are candidates for ICB therapy (Duffy and Crown, 2019). A large number of studies utilized the survival data of patients who received ICB treatment to identify the characteristics of patients with good survival. These studies sought to identify a biomarker consisting of particular genes or construct a signature based on mRNA expression profiles or mutation data of genes that could accurately represent the response to ICB (Patel and Kurzrock, 2015; Hugo et al., 2016; Goodman et al., 2017; Jamieson and Maker, 2017; Panda et al., 2017; Chang et al., 2018; Cristescu et al., 2018; Miao et al., 2018; Duffy and Crown, 2019; Samstein et al., 2019; Bai et al., 2020; Zhou et al., 2020; Jiao et al., 2021; Pan et al., 2021). Most of the signatures were constructed for specific cancers, such as melanoma and non-small cell lung cancer (NSCLC) (Bai et al., 2020; Zhou et al., 2020; Jiao et al., 2021; Pan et al., 2021). For pancancer, high TMB (TMB-H) [≥10 muts/Mb] has been approved to be a biomarker for ICB selection by the FDA (Food and Drug Administration, 2021). In fact, the determination of TMB requires the assessment of mutational features of hundreds of genes. TMB treats mutational information for genes equally and simply focuses on the number of mutations. Thus, TMB might not represent a cost-effective signature. Therefore, it is critical to find a more effective and precise signature for ICB treatment.

In this study, we focused on predicting the pancancer response of patients to ICB with agents, such as anti-programmed cell death protein 1 (PD-1), anti-programmed death-ligand 1 (PD-L1), and/or anti-cytotoxic T-lymphocyte-associated protein 4 (CTLA-4). We proposed a framework to construct a signature with a small number of genes. The signature was expected to have a better predictable performance on ICB-treated datasets than TMB. To better demonstrate the performance of the proposed signature, we performed multicohort validation. Furthermore, an association analysis of tumor-infiltrating lymphocytes (TILs) was conducted.

## Materials and methods

### Clinical cohorts

As mentioned above, we conducted a multicohort validation. Specifically, in our study, we used two types of cohorts: ICB-treated datasets and non-ICB-treated datasets. For ICB-treated datasets, we used two immunotherapy cohorts from Memorial Sloan-Kettering Cancer Center (MSKCC) and Dana-Farber Cancer Institute (DFCI), termed MSK-TMB and ALLEN, respectively (Miao et al., 2018; Samstein et al., 2019). Non-ICB-treated datasets included two nonimmunotherapy cohorts from MSKCC and TCGA (named as MSK-IMPACT and TCGA). All data were collected from previously published clinical cohorts. We obtained MSK-TMB, ALLEN, and MSK-IMPACT from the cBioPortal database (https://www.cbioportal.org) (Cerami et al., 2012) and obtained Pan-Cancer Atlas Hub of TCGA data from UCSC Xena (https://xenabrowser.net/datapages/) (Goldman et al., 2020). We randomly partitioned 80% and 20% of the samples from MSK-TMB into a training set and a test set named MSK-TMB-training and MSK-TMB-test, respectively. For the analysis of the tumor immune microenvironment (TiME), we obtained infiltration estimations for all TCGA tumors from http://timer.cistrome.org, which included the results of the CIBERSORT algorithm (Newman et al., 2015; Li et al., 2017). GEPIA (http://gepia.cancer-pku.cn) was used to analyze the gene mRNA expression levels in tumors compared with paired normal tissues. Information regarding protein expression of cell lines and tissues was obtained from the Human Protein Atlas (HPA, https://www.proteinatlas.org). We also used the TISIDB database (http://cis.hku.hk/TISIDB) to infer the relationship between the abundance of TILs and DNA methylation.

After removing patients with missing survival times, the MSK-TMB-training, MSK-TMB-test, and ALLEN datasets consisted of 1329, 332, and 249 samples, respectively. MSK-IMPACT and TCGA had 6256 and 8724 samples, respectively. The MSK-TMB-test and ALLEN datasets represent two ICB-treated cohorts from different centers for validation. The mutations (1 for mutated and 0 for wild type) of 468 cancer-related genes from the MSK-TMB-training cohort were considered as candidate features for constructing a signature. The overall survival (OS) time was the target of our statistical model. More details about the clinical characteristics of the five datasets are provided in online supplementary Table S1.

### Construction of a signature framework and statistical analysis

We developed a study framework to construct a signature for predicting the response to ICB treatment. We learned a signature based on MSK-TMB-training and then validated the signature on the other four datasets. The signature was constructed based on gene feature selection and weighting, and the candidate features for the work were mutational data of hundreds of genes.

Because genes mutated with extreme-low frequency were unable to provide reliable information for the predictable task, we only considered genes with mutation frequency ≥1%. Then, we selected genes for which their mutations might represent potential factors influencing patient survival. Specifically, we conducted a univariate Cox proportional hazards regression analysis for each gene, and genes with a *p* value ≤ 0.05 were selected for the next step. Given that only a few genes among the selected genes that truly impact survival, we conducted a multivariate analysis to achieve both gene selection and signature generation.

We built a multivariate Cox proportional hazards regression model with the least absolute shrinkage and selection operator (LASSO), and the features included the mutational status of the selected genes (Tibshirani, 1996; Huang et al., 2016; Reichling et al., 2020). LASSO is powerful in selecting the key features and avoiding model overfitting. Specifically, based on Cox regression, LASSO introduced a penalty item with a hyperparameter on feature variables, and the choice of the hyperparameter impacted the result of feature selection. A large numeric hyperparameter might lead to an overpenalty on feature selection, causing few features to remain and the model to be underfit. In contrast, a low numeric hyperparameter might lead to a weak effect on feature selection. Thus, we conducted 5-fold cross-validation to determine the hyperparameter. Since the concordance index (C-index) evaluated the predicted performance of a Cox regression model, we could decide on a hyperparameter based on the C-index. The model with fewer features (genes in our study) and satisfactory C-index value should be preferred. Therefore, our recommendation strategy was that we should determine the hyperparameter considering both the C-index and the number of remaining features, rather than maximizing the C-index. After the penalty hyperparameter was determined, a Cox model with LASSO was built. The risk score of the model, which is derived from a weighted sum of gene mutations, is the desired signature.

Kaplan–Meier survival curves were generated to intuitively display the survival of patients in groups. The log-rank test was used to analyze the differences in survival among the different patient groups. A 95% confidence interval for median OS was reported. All statistical analyses were performed in R language (version 4.1.0). Unless stated otherwise, all *p*-values were two-sided with an *α* of 0.05.

## Results

### Development of a potential immune predictive gene signature of signature of pancancer

The use of potential gene mutations as biomarkers could functionally affect the outcome of immunotherapy. Starting with 468 genes, we constructed a signature with 18 genes. There were 288 genes with mutational frequency ≥1% in MSK-TMB-training. After univariate Cox proportional hazards regression analysis, 109 genes with statistical significance remained (*p* ≤ 0.05). We performed 5-fold cross-validation to decide an appropriate penalty hyperparameter *λ* of the Cox regression model with LASSO. Supplementary Figure S1 shows the association between penalty hyperparameter *λ* and the C-index. Considering the trend of the association between the C-index and the number of the remaining genes, we took *λ* = 0.0575 and built a Cox regression model with LASSO using the hyperparameter. Finally, we determined the Cox regression model with LASSO, which included 18 genes. We named the risk score the SIGP (signature of pancancer) score. The desired risk score formula from the model was defined as follows:

$$\begin{array}{l} SIGP\;score=-0.3485^{*}VHL-0.2073^{*}TET1-0.1501\\ {\;}^{*}FAM46C-0.1294^{*}NTRK3-0.1006^{*}BRAF-0.0986\\ {\;}^{*}NOTCH3-0.0983^{*}RNF43-0.0807^{*}TERT-0.077^{*}\\ PTPRT-0.0582^{*}LATS1-0.0509^{*}SETD2-0.0448\\ {\;}^{*}PAK7-0.0381^{*}PTPRD-0.0366^{*}IL7R-0.0192\\ {\;}^{*}ROS1-0.0178^{*}CREBBP+0.0908^{*}STK11+0.1304^{*}TP53 \end{array}$$

**FIGURE 1:**
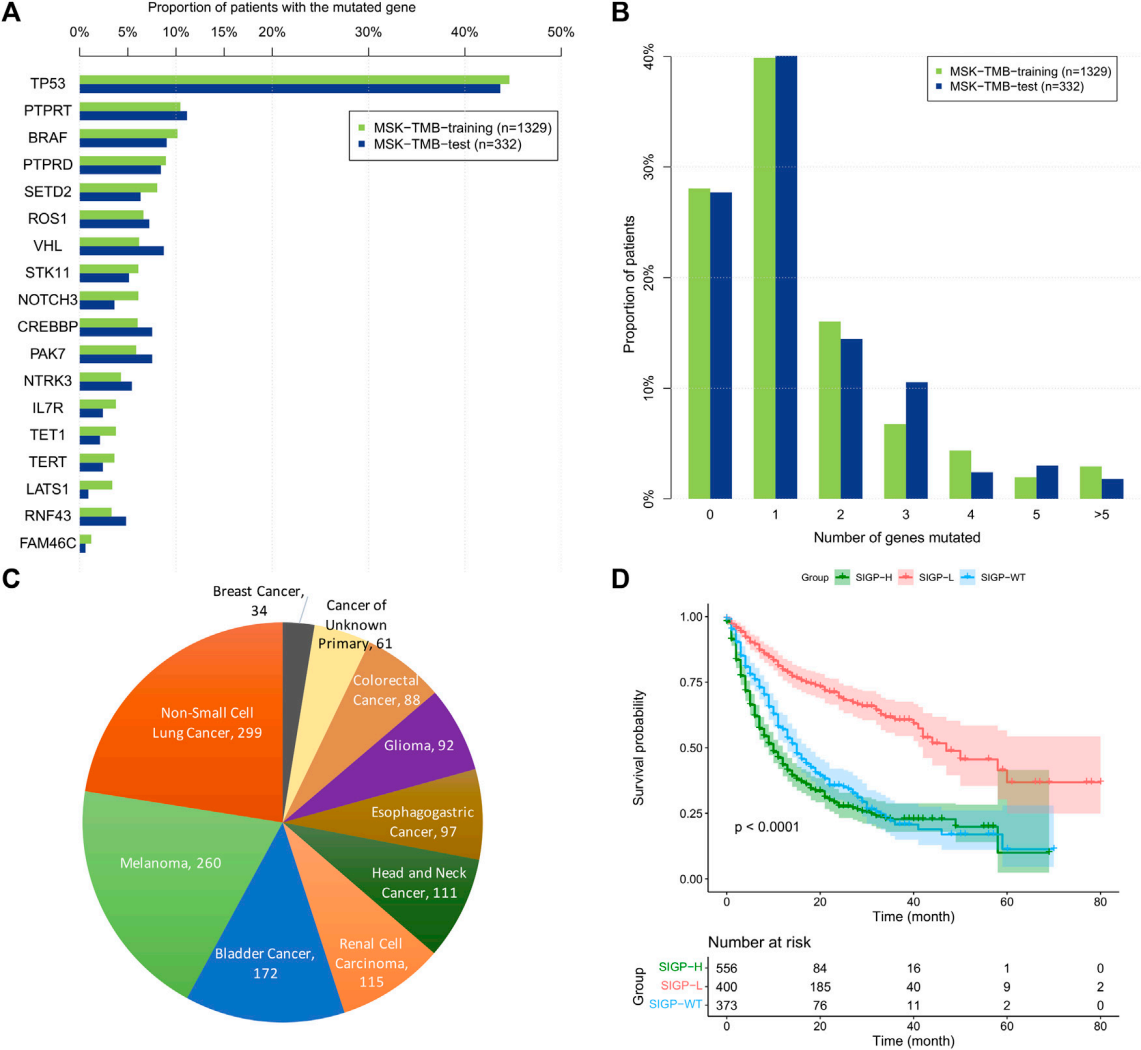
The prevalence of 18 selected genes mutated among patients and the proportion of patients with the different number of genes mutated in the MSK-TMB-training cohort and the MSK-TMB-test cohort, the proportion of patients with different cancer types and Kaplan–Meier (KM) curves of SIGP in MSK-TMB-training. **(A)** Prevalence of the 18 selected genes mutated among patients; **(B)** Proportion of patients with the different number of genes mutated; **(C)** Proportion of patients with different cancer types in MSK-TMB-training (*n* = 1329); **(D)** KM curves of SIGP in MSK-TMB-training.

For the 18 selected genes, Figures 1A–C shows the prevalence of the genes mutated among patients and the proportion of patients with different number of genes mutated in the MSK-TMB-training cohort and the MSK-TMB-test cohort, and proportion of patients with different cancer types in the MSK-TMB-training cohort. Most of the 18 genes of SIGP corresponded to a prevalence greater than 5%, and SIGP was available for providing a predicted result of the response to ICB for a large proportion of patients. However, we should note that approximately 28% of patients did not harbor any mutations among the 18 genes. We included patients with no mutations among 18 genes as a group, i.e., SIGP-WT. We used the maximally selected rank statistics based on the SIGP score and OS for patients with mutated genes in the training cohort to determine the optimal cutoff point. Then, we separated patients into three groups: SIGP-WT, SIGP-L (≤−0.0212241), and SIGP-H (>−0.0212241 & ≠ 0). As a result, 373 (28.07%), 400 (30.10%), and 556 (41.83%) of 1329 patients in the MSK-TMB-training dataset were classified into the SIGP-WT, SIGP-L, and SIGP-H groups, respectively, and their median OS values were 15 (13–17), 47 (42-NA), and 10 (9–12) months, respectively. Patients with low SIGP obtained significantly longer OS than the other patients (*p* < 0.0001). Figure 1D shows the performance of SIGP in the training set.

### Validation of signature of pancancer on the immune checkpoint blockade-treated cohorts

**FIGURE 2:**
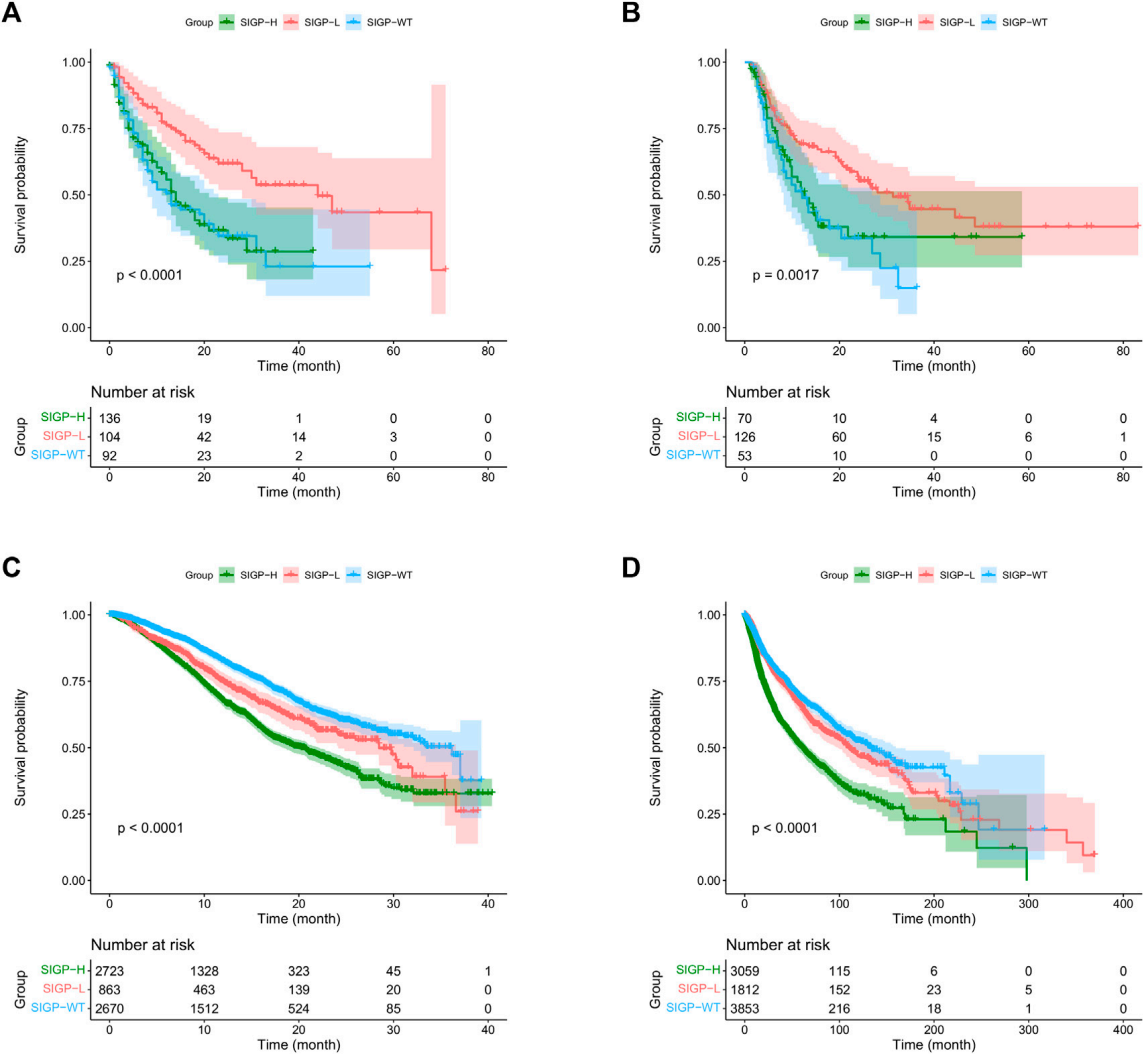
Kaplan–Meier (KM) curves of SIGP in two ICB-treated cohorts and two non-ICB-treated cohorts: MSK-TMB-test, ALLEN, MSK-IMPACT and TCGA. **(A)** KM curves of SIGP in MSK-TMB-test; **(B)** KM curves of SIGP in ALLEN; **(C)** KM curves of SIGP in MSK-IMPACT; **(D)** KM curves of SIGP in TCGA.

We validated the performance of SIGP in predicting the efficacy of ICB treatment in the MSK-TMB-test cohort. To demonstrate the generalization of SIGP, we validated ALLEN, which is an ICB-treated cohort from a different center. We calculated the SIGP scores of patients of the MSK-TMB-test and ALLEN and separated patients into three groups based on the determined cut-point (−0.0212241) and the SIGP scores. The validation result shows a general pattern of SIGP: patients with SIGP-L had a significantly longer OS than patients with SIGP-WT and SIGP-H (Figures 2A,B). The median OS values of SIGP-WT, SIGP-L, and SIGP-H in the MSK-TMB-test were 13.00 (8.00–23.00), 44.00 (28.00-NA), and 14.00 (11.00–21.00) months, respectively, while those in ALLEN were 12.11 (8.22–26.91), 31.32 (23.82-NA), and 12.63 (9.61-NA) months, respectively. In particular, a large proportion of patients with longer OS (e.g., >20.00 months) were identified in SIGP-L. As a result, SIGP-L was determined to represent a potential signature of a good response to ICB treatment.

### Performance of signature of pancancer on the non-immune checkpoint blockade-treated cohorts

We conducted an experiment to verify whether SIGP represents a specific signature for ICB treatment without the independent influence of prognostic factors. Thus, we validated the performance of SIGP on the non-ICB-treated cohorts and compared the results between non-ICB-treated cohorts and ICB-treated cohorts. We computed the SIGP scores of patients in two non-ICB-treated cohorts and then obtained the SIGP-WT, SIGP-L, and SIGP-H groups (Figures 2C,D). Kaplan–Meier survival curves of OS were generated for three groups of patients from the MSK-IMPACT and TCGA datasets. Compared with the results of the ICB-treated cohorts (Figures 2A,B), we observed the following: 1) Patients in the SIGP-L group did not exhibit the best prognosis, and their survival was significantly lower than that of the SIGP-WT group (log-rank test for SIGP-WT and SIGP-L: MSK-IMPACT, *p* < 0.0001; TCGA, *p* = 0.0428). 2) It is obvious that patients in the SIGP-WT group had significantly longer OS than patients in the SIGP-H group. These findings were different from the results of the ICB-treated cohorts. Therefore, SIGP generated from ICB-treated cohorts plays a potential specific role in predicting the response to ICB treatment and was not able to be generalized to non-ICB-treated cohorts.

### Performance of signature of pancancer against tumor mutation burden

As mentioned above, TMB-H was approved as one of the biomarkers for ICB treatment by the FDA. We compared the performance of SIGP-L with that of TMB-H in the MSK-TMB-test cohort (Figure 2A and Supplementary Figure S2). We observed that SIGP had a similar performance to TMB. However, SIGP is based on only 18 genes and recognized more patients with longer OS, whereas TMB is based on hundreds of genes. Thus, our signature SIGP is cost-effective. Here, we explored the effects of the combination of SIGP and TMB. As demonstrated above, low SIGP and high TMB indicated a good response to ICB therapy. Consistent with our expectation, the combination of SIGP-L and TMB-H exhibited the best survival result, whereas the combination of SIGP-H and TMB-L corresponded to the worst survival curve (Supplementary Figure S3). In addition, we observed that the SIGP-L + TMB-L group shows a good prognosis for ICB therapy. Table 1 shows the proportions and median survival of the three groups: SIGP-L + TMB-H, SIGP-L + TMB-L, and SIGP-H + TMB-L. Approximately 30% of patients in the cohorts SIGP-L + TMB-H and SIGP-L + TMB-L were predicted to benefit from the ICB treatment, whereas an additional 30% of patients, i.e., SIGP-H + TMB-L might not be recommended to accept ICB treatment.

**TABLE 1:** Proportions (Prop.) and median survival of SIGP-L + TMB-H, SIGP-L + TMB-L, SIGP-H + TMB-L and Others in the MSK-TMB-test cohort and the MSK-TMB-training cohort.

Group	MSK-TMB-test		MSK-TMB-training	
	Prop	Median OS (95% CI)	Prop	Median OS (95% CI)
SIGP-L + TMB-H	49/332 (14.76%)	NA (31-NA)	223/1329 (16.78%)	60 (44-NA)
SIGP-L + TMB-L	55/332 (16.57%)	44 (21-NA)	177/1329 (13.32%)	36 (32-NA)
SIGP-H + TMB-L	107/332 (32.23%)	12 (8–18)	392/1329 (29.50%)	9 (8–11)
Others	121/332 (36.45%)	18 (12–31)	537/1329 (40.41%)	14 (13–16)

**FIGURE 3:**
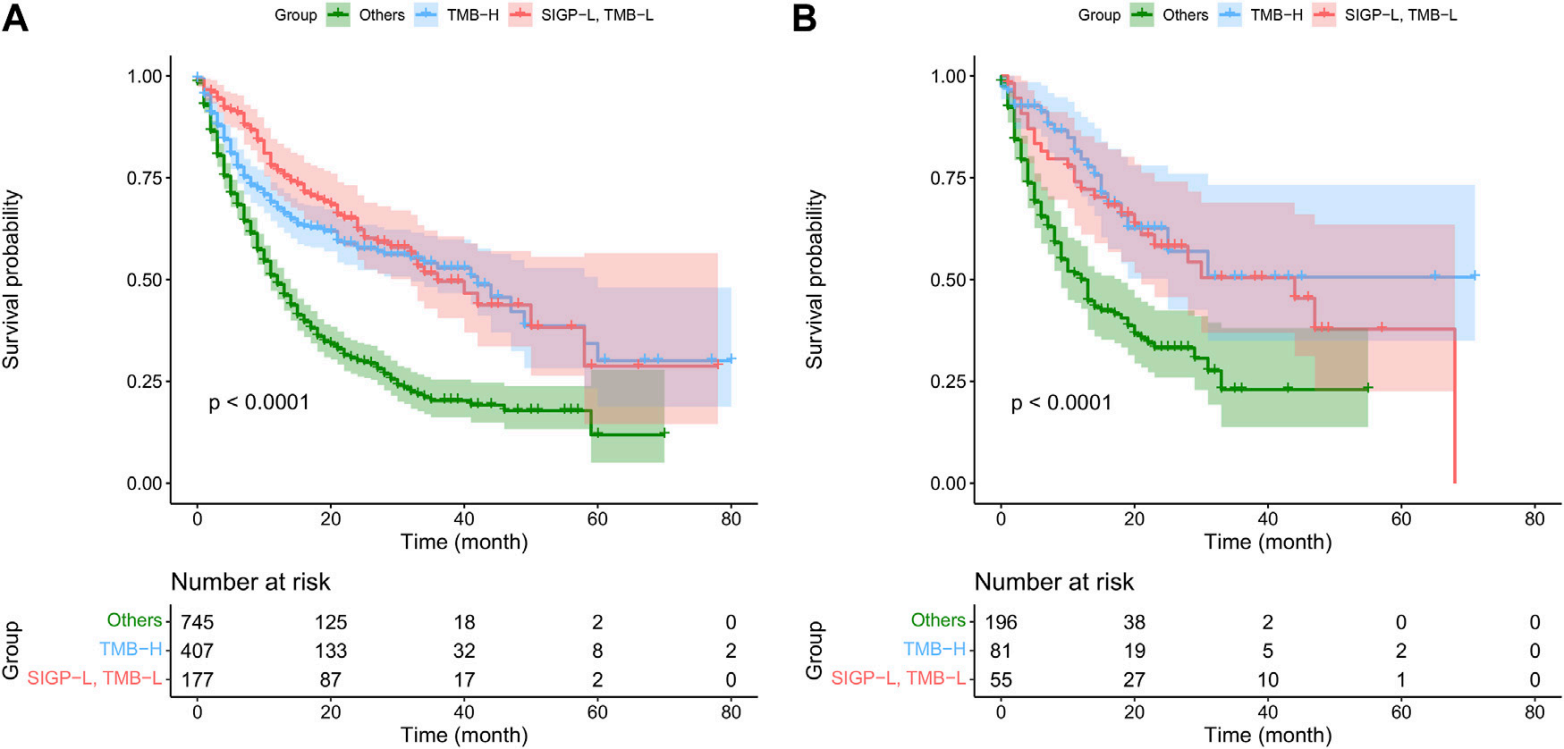
Kaplan–Meier curves of TMB combined with SIGP (three groups) in MSK-TMB-training and MSK-TMB-test. **(A)** KM curves of SIGP + TMB in MSK-TMB-training; **(B)** KM curves of SIGP + TMB in MSK-TMB-test.

Furthermore, we stratified patients into three groups: TMB-H, SIGP-L + TMB-L, and others. The median OS in the MSK-TMB-test was NA (25-NA), 44 (21-NA), and 12 (9–18) for the TMB-H, SIGP-L + TMB-L, and others groups, respectively, and the median OS in the MSK-TMB-training was 42 (32–60), 36 (32-NA), and 12 (11–14), respectively. Figure 3 displays the KM curves of the three groups. The SIGP-L + TMB-L group had similar survival to the TMB-H group. Thus, our signature SIGP identified extra candidates (16.57% in MSK-TMB-test, 13.32% in MSK-TMB-training) for ICB treatment in addition to the patients with TMB-H.

Overall, the combination of SIGP and TMB could improve the predictive value and increase the confidence of identifying patients with a positive or negative response to ICB treatment. An additional approximately 15% of patients with low TMB were identified as candidates for ICB treatment by the SIGP.

### Association between signature of pancancer and tumor immune microenvironment

**FIGURE 4:**
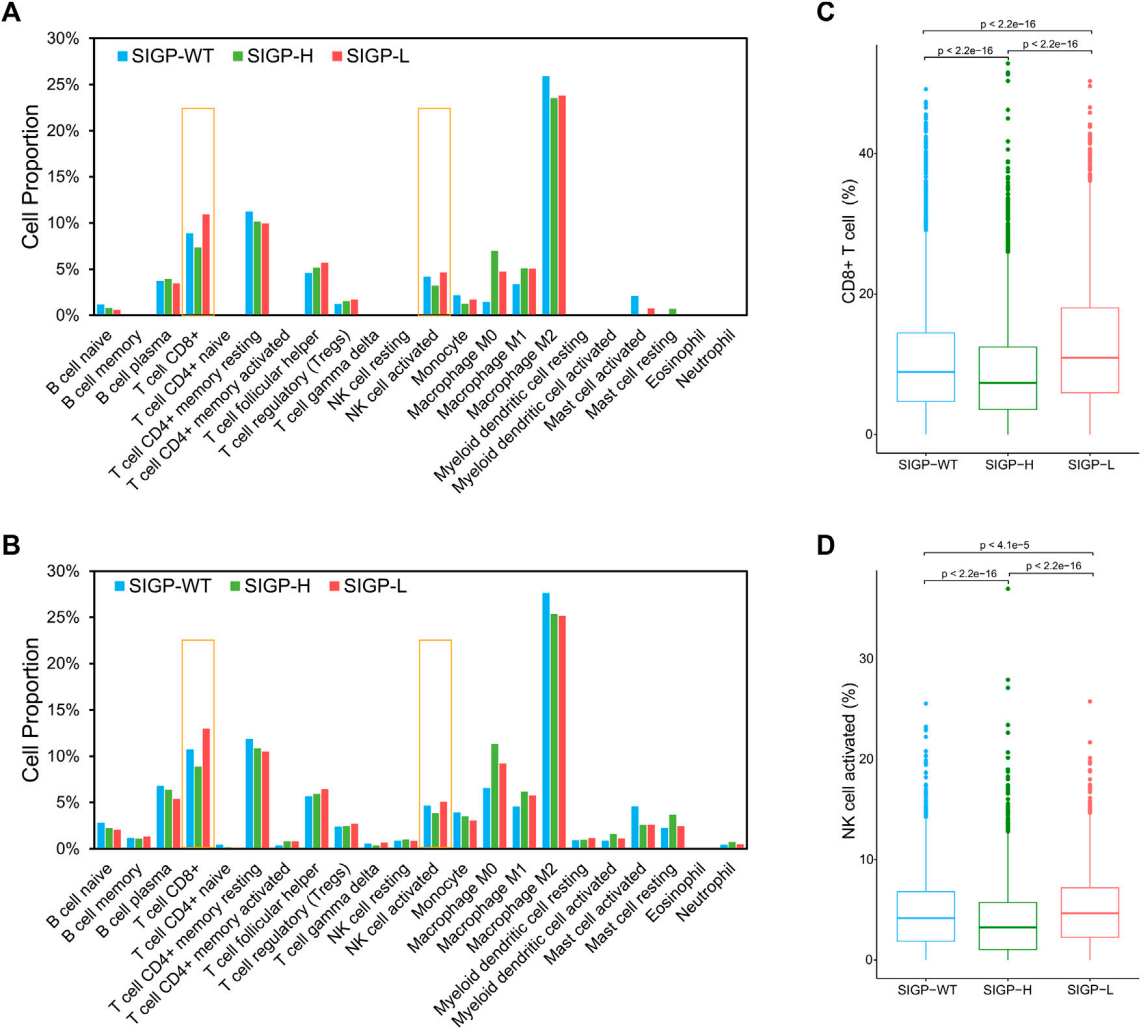
Medians and means of CIBERSORT estimates of abundances in a mixed cell population for 22 TIL cell types and boxplots of CIBERSORT estimates of abundances for CD8^+^ T cells and NK cell activated in the TCGA cohort. **(A)** Medians of CIBERSORT estimates of abundances for immune cell types; **(B)** Means of CIBERSORT estimates of abundances for immune cell types; **(C)** Boxplot for CD8^+^ T cells (Wilcoxon test); **(D)** Boxplot for NK cell activated (Wilcoxon test).

TiME, an important effector battlefield for immunotherapy, has been shown to correlate with the efficacy of immunotherapy in numerous studies (Ock et al., 2016; Varn et al., 2017; Bagaev et al., 2021). We obtained CIBERSORT estimates of abundances of 22 immune cell types for 8724 TCGA cohort patients and observed the differences between the three groups separated by SIGP to assess the association between SIGP and TiME. Figures 4A,B illustrate medians and means of CIBERSORT estimates of abundances of 22 immune cell subtypes. Significant differences in the abundances of CD8^+^ T cells, M0 macrophages, activated NK cells, and especially CD8^+^ T cells were noted between SIGP-L and SIGP-H. Figures 4C,D shows boxplots of CIBERSORT estimates of the abundances of CD8^+^ T cells and activated NK cells. Compared with SIGP-H and SIGP-WT, SIGP-L had a higher proportion of CD8^+^ T cells and activated NK cells, which would play a crucial role in eliminating cancer cells.

### Expression of genes involved in signature of pancancer

Gene mutations may affect protein expression and function, and we additionally assessed the mRNA and protein expression levels of the most important genes in SIGP [top2 in positive contribution for SIGP score (*TP53, STK11*) and top4 genes in negative contribution for SIGP score (*VHL, TET1, FAM46C*, and *NTRK3*)] in our study in cell lines and human tissues from public databases, including TCGA and HPA (Supplementary Figure S4). The results showed that these genes were expressed differently in different types of cell lines and tissues. STK11 and VHL were widely expressed at the mRNA and protein levels in various cell lines and tissues, whereas TP53 was more sensitively detected at the mRNA level. We also explored the differences between these genes in tumor tissues and normal tissues, which may explain the potential basis of this model (Supplementary Figure S5). The expression levels of oncogenes or tumor-related genes, such as BRAF, NOTCH3, and TERT, were higher in tumor tissues compared with paired normal tissues. Tumor suppressor genes TP53 and VHL also showed a tendency to be more highly expressed in tumor tissues than in normal tissues. We further examined the relationship between DNA methylation and the immune microenvironment, and the results showed that the methylation of several important genes in this model (*TP53, STK11* and *VHL*) may be associated with a better immune microenvironment (Supplementary Figure S6). The current results make it difficult to explain whether DNA methylation is related to the model in this study, and further data are needed to clarify this issue.

## Discussion

Immunotherapy, especially ICB treatment, is a breakthrough in antitumor therapy and has revolutionized outcomes in advanced cancers. However, ICB therapy might cause adverse effects, and only a proportion of patients could benefit from it. It is crucial to have biomarkers to accurately predict the efficacy of ICB treatment and support treatment decisions for patients. Our work conducted a multicohort and retrospective study to develop a potential pancancer signature to predict ICB therapy efficacy.

We conducted multiple experiments to verify the performance of our proposed 18-gene-based SIGP signature, and the validation results of SIGP in two ICB-treated cohorts from different centers and two non-ICB-treated cohorts demonstrated that the signature captured a general pattern of predicting the response to ICB. The comparison experiment between SIGP and TMB showed that SIGP could recognize more patients with a longer survival time. The analysis on the merging of SIGP and TMB revealed a better result: patients with two positive signs for ICB, i.e., SIGP-L and TMB-H, had a higher survival probability. in contrast, most of the patients with SIGP-H and TMB-L had a shorter OS and a lower survival probability. Thus, SIGP-L + TMB-H and SIGP-H + TMB-L potentially represent positive and negative ICB treatment efficacy, respectively. In addition, SIGP-L + TMB-L performed similarly to TMB-H in terms of survival in approximately 15% of patients. Thus, the discovery ability of TMB-H in patients who responded to ICB was limited, and SIGP-L identified a few responders ignored by TMB-H.

Our framework selected key genes during statistical modeling. As mentioned above, the computation of TMB is based on hundreds of genes with the same level of importance. Our framework identified 18 key genes and assigned them with different importance values (see the coefficients) to represent varying impacts. In fact, gene selection was performed three times in our network. First, we removed genes with a prevalence of mutations less than a given threshold. Second, we conducted a univariate analysis to determine the impact of gene mutations on the survival of patients and selected genes with significant influences. Finally, a multivariate Cox regression with LASSO was built where the penalty hyperparameter was determined by considering the associations between the C-index and the number of the remaining genes after a 5-fold cross-validation experiment. The LASSO method reduces the variance of the predicted values by shrinking the coefficients without a substantial increase in the bias, which means that LASSO could drop many irrelevant features that are not associated with the response variable. As a result, the LASSO method could reduce overfitting and improve the predictive performance of models, especially when the number of features is large. The LASSO method is powerful and widely used in multiple tasks, such as high-dimensional cancer classification, genomic selection, and prediction tasks (Wu et al., 2009; Ogutu et al., 2012; Algamal and Lee, 2015; Ranstam and Cook, 2018; Xiong et al., 2019).

The immune subsets in the TiME were recently recognized to be closely involved in the response of ICB treatment. It is interesting to explore the associations between SIGP and the TiME. Our further TCGA cohort analyses demonstrated that SIGP was associated with higher CD8^+^ T cells in the TiME. Existing evidence has confirmed that CD8^+^ T cells are the most powerful effectors in the antitumor immune response, and ICB treatment works to block suppressive immune receptors and then restore the functions of T cells, including CD8^+^ T cells (Savage et al., 2008; Mahmoud et al., 2011; Hadrup et al., 2013; Raskov et al., 2021). Specifically, a study of various cancers showed that the abundance of CD8^+^ T cells in a tumor was the best predictive factor for the efficacy of anti-PD-1/PD-L1 therapy (Lee and Ruppin, 2019). Actually, the coefficients of the selected genes in SIGP indicated their impacts on ICB efficacy and were associated with the basis of SIGP scoring. Based on the coefficients of the 18 genes, two groups of genes, which corresponded with positive or negative coefficients, were identified. Given that patients with a low SIGP score had better survival than others, genes with positive coefficients should be expected to be tumor suppressor genes, and mutations in these genes negatively affect the efficacy of ICB. On the one hand, STK11 and TP53 were the only two coefficients with a positive value, indicating that mutations in these genes would lead to a poor response to ICB treatment in our study. This result was consistent with the fact that STK11 and TP53 are two tumor suppressor genes and that their mutations are known as negative prognostic effectors of ICB treatment (Schabath et al., 2016; Pécuchet et al., 2017). On the other hand, mutations in the other 16 genes with negative coefficients contributed to a good response to ICB treatment, and these genes seemed to be associated with better immunogenicity and immunomodulatory effects. RNF43 was identified as a tumor-associated antigen that can lead to tumor-reactive cytotoxic T-cell responses (Uchida et al., 2004), and a VHL mutation was associated with outcomes and promoted increased NK cell infiltration (Perier et al., 2011), explaining the increased number of CD8^+^ T cells and NK cells in patients with low SIGP scores in this study. Mutations in TET1, a DNA demethylase, were enriched in responders to ICB and were strongly associated with enhanced tumor immunogenicity and a relatively hot immune microenvironment (Wu et al., 2019). Mutations in PTPRD, CREBBP, BRAF, NOTCH3, TERT, and SETD2, which are involved in various aspects of immune regulation, were reported as potential biomarkers in cancer patients after immunotherapy with improved survival (Ackerman et al., 2014; Ortiz et al., 2014; Li et al., 2020; Lin et al., 2020; Lu et al., 2021; Yan et al., 2021; Zhang et al., 2022). Cells with NTRK3 mutations are more immunogenic (Niu et al., 2020; Zhang et al., 2021), and LATS1 mutations may lead to considerable immune reprogramming (Moroishi et al., 2016). Therefore, the genes in SIGP might affect the response to ICB treatment positively or negatively. And, patients categorized into SIGP-L based on immune-related genes had a higher abundance of CD8^+^ T cells compared with the other patients, explaining why SIGP-L patients had a better prognosis in response to ICB treatment.

The patient distribution of the training cohort had an impact on our predictive work. Here, our discussion focused on the types of cancer among patients. First, we should note that our predictive work did not consider any information about cancer types. Figure 1C shows the proportions of the cancer types in the training cohort, which might affect the selection of genes related to immunotherapy. There were numerous studies about the associations between the selected 18 genes and the cancer types. TET1, TERT, and SETD2 mutations represent potential biomarkers of ICB treatment in multiple cancers (Wu et al., 2019; Li et al., 2020; Lu et al., 2021). NTRK3 mutation might contribute to a good prognosis of ICB in NSCLC and bladder cancer (Niu et al., 2020; Zhang et al., 2021). VHL mutation was associated with the better survival of renal cell carcinoma patients (Kim et al., 2005; Patard et al., 2008; Kammerer‐Jacquet et al., 2017). TERT, BRAF, NOTCH3, and PRPRT mutations were associated with the response in melanoma patients (Ackerman et al., 2014; Li et al., 2020; Yan et al., 2021; Zhang et al., 2022). Furthermore, different cancer types might different survival times in response to immunotherapy. Thus, a few cancer types might have a relatively long survival time, such as renal cell cancer, whereas a few cancer types corresponded to a relatively short overall survival, such as breast cancer. The aim of our work was to predict a longer overall survival in the context of ICB treatment, and our validation result demonstrated that our SIGP signature had a good predictive performance. Because particular cancer types might have a longer survival time than other cancer types, our work might classify patients with cancer types with relatively longer OS into the long-surviving group. The differences in the survival of patients with different particular cancer types might be much larger than those with the same cancer type. Thus, our predictive work might give more attention to the differences between cancer types rather than the differences in patients with the same cancer type. However, stratifying long-surviving patients with a particular cancer type is an important task. This issue could potentially be solved by incorporating the information of cancer types or targeting a single cancer type, representing aims of our future studies.

The performance of SIGP was relative to the patient distribution of validation cohorts. The MSK-TMB-test cohort followed the same patient distribution as noted in the training cohort, and the performance of SIGP in the MSK-TMB-test was similar to that noted in the training cohort. As an extra validation dataset, ALLEN had a different patient distribution (Supplementary Table S1), and its SIGP performance was not as good as that of the MSK-TMB-test. We need to consider the distribution of cancer types to pursue better performance in the prediction of efficacy to ICB treatment in multiple cancers.

The comparison of the performance of SIGP between the ICB-treated cohorts and the non-ICB-treated cohorts also demonstrated that our predictive work selected the key genes with mutations related to ICB treatment. Our validation result shows that SIGP-WT in the non-ICB-treated cohorts had the best survival performance compared with the other two groups, which was consistent with biological knowledge. For non-ICB treatment, gene mutations might cause aggressive tumors or drug resistance, so patients with longer OS might have wild-type genes.

There are several limitations of our work in addition to those mentioned above. First, only a few patients with short survival were classified into the SIGP-L group. Thus, our SIGP model is not perfect in precisely identifying responders to ICB. In fact, screening all responders is difficult and challenging. Considering more information is one method to improve the performance of the predictive model. Our work was based on the mutational status (1/0) of 468 genes and ignored the number of mutations and specific variant types of a gene. In fact, considering too many features increases the number of samples required. Even when considering the mutational status of 468 genes, there may be numerous combinations of genes that serve as patterns for signatures of ICB efficacy. However, the sample size of the training dataset for our predictive task is too small to provide sufficient information for models to identify an optimal pattern. Regarding model structure, two limitations of Cox regression with LASSO are noted. One limitation is that LASSO might select one feature randomly in the case there are two or more highly collinear variables. Another limitation is that the regression is based on the assumption of the linear relationship between features and log hazard ratio, which might not be logical when the relationship is nonlinear. It is challenging to address these issues, and it would be our future work.

In conclusions, our work proposed a study framework for constructing a genetic mutation signature predictive of ICB treatment response. The SIGP signature is cost-effective and performed well in predicting the response to ICB. SIGP was associated with CD8^+^ T cells. Specifically, SIGP-low patients who benefited from ICB exhibited an increased abundance of CD8^+^ T cells. Compared with TMB, SIGP potentially identified more patients who would benefit from ICB. The combination of SIGP and TMB improved the predictive value of the efficacy of ICB and identified a large proportion of patients with a positive or negative response to ICB. Approximately 15% of patients without TMB-H were identified as candidates of ICB therapy by SIGP. Furthermore, our framework provided a potential solution for solving a similar task.

## Data Availability

The original contributions presented in the study are included in the article/Supplementary Material, further inquiries can be directed to the corresponding author.
