# Supplementary material for: Pancancer analysis of a potential gene mutation model in the prediction of immunotherapy outcomes
Source: Front Genet. 2022 Aug 26;13:917118. doi: 10.3389/fgene.2022.917118 (PMC9459043; doi:10.3389/fgene.2022.917118)
Supplement: Supplementary file 1 [file Table1.DOCX]

Supplementary Materials

**Table S1.** Clinical characteristics of patients in five cohorts.

| Cohort | No. pts | #(Alive status) | #(Death status) | Median of OS (months) | The 3 most frequent cancers | | |
| --- | --- | --- | --- | --- | --- | --- | --- |
| MSK-TMB-training | 1329 | 654 (49.21%) | 675 (50.79%) | 11.00 | NSCLC (22.50%) | Melanoma (19.56%) | Bladder (12.94%) |
| MSK-TMB-test | 332 | 175 (52.71%) | 157 (47.29%) | 10.00 | Melanoma (18.07%) | NSCLS (15.36%) | Bladder (12.95%) |
| ALLEN | 249 | 124 (49.80%) | 125 (50.20%) | 11.84 | Melanoma (60.64%) | NSCLS (22.49%) | Bladder (10.84%) |
| MSKIMPACT | 6256 | 4467 (71.40%) | 1789 (28.60%) | 10.55 | NSCLC (17.62%) | Breast (12.18%) | Colorectal (9.40%) |
| TCGA | 8724 | 6260 (71.76%) | 2464 (28.24%) | 23.57 | BRCA (9.02%) | LGG (5.83%) | LUAD (5.73%) |

**Figure S1.** The 5-fold cross validation results (C-index) of Cox regression models with LASSO for different penalties λ where a point corresponds to a C-index value and the number of selected features (see the number above the figure frame) resulting from a Cox regression model with LASSO with a particular λ.

|   **(A)** |   **(B)** |
| --- | --- |

**Figure S2.** Kaplan–Meier curves of TMB and SIGP in MSK-TMB-test. (**A**) KM curves of TMB in MSK-TMB-test; (**B**) KM curves of TMB and SIGP in MSK-TMB-test.

|   **(A)** |   **(B)** |
| --- | --- |

**Figure S3.** Kaplan–Meier curves of TMB combined with SIGP (four groups) in MSK-TMB-training and MSK-TMB-test. **(A)** KM curves of SIGP+TMB in MSK-TMB-training; **(B)** KM curves of SIGP+TMB in MSK-TMB-test.


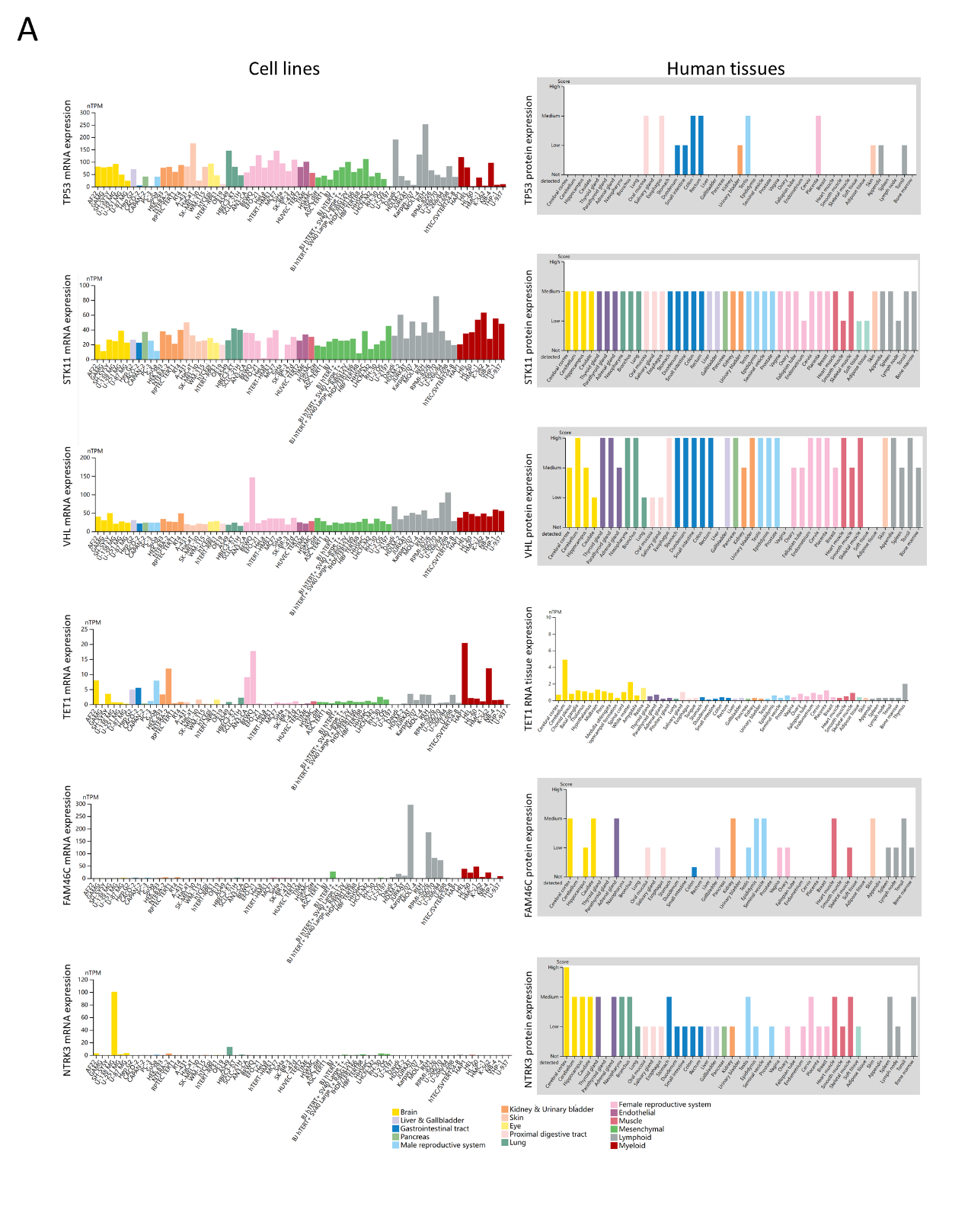


**Figure S4.** Expression level of mRNA and protein of the key genes in our study in cell lines and human tissues by HPA.


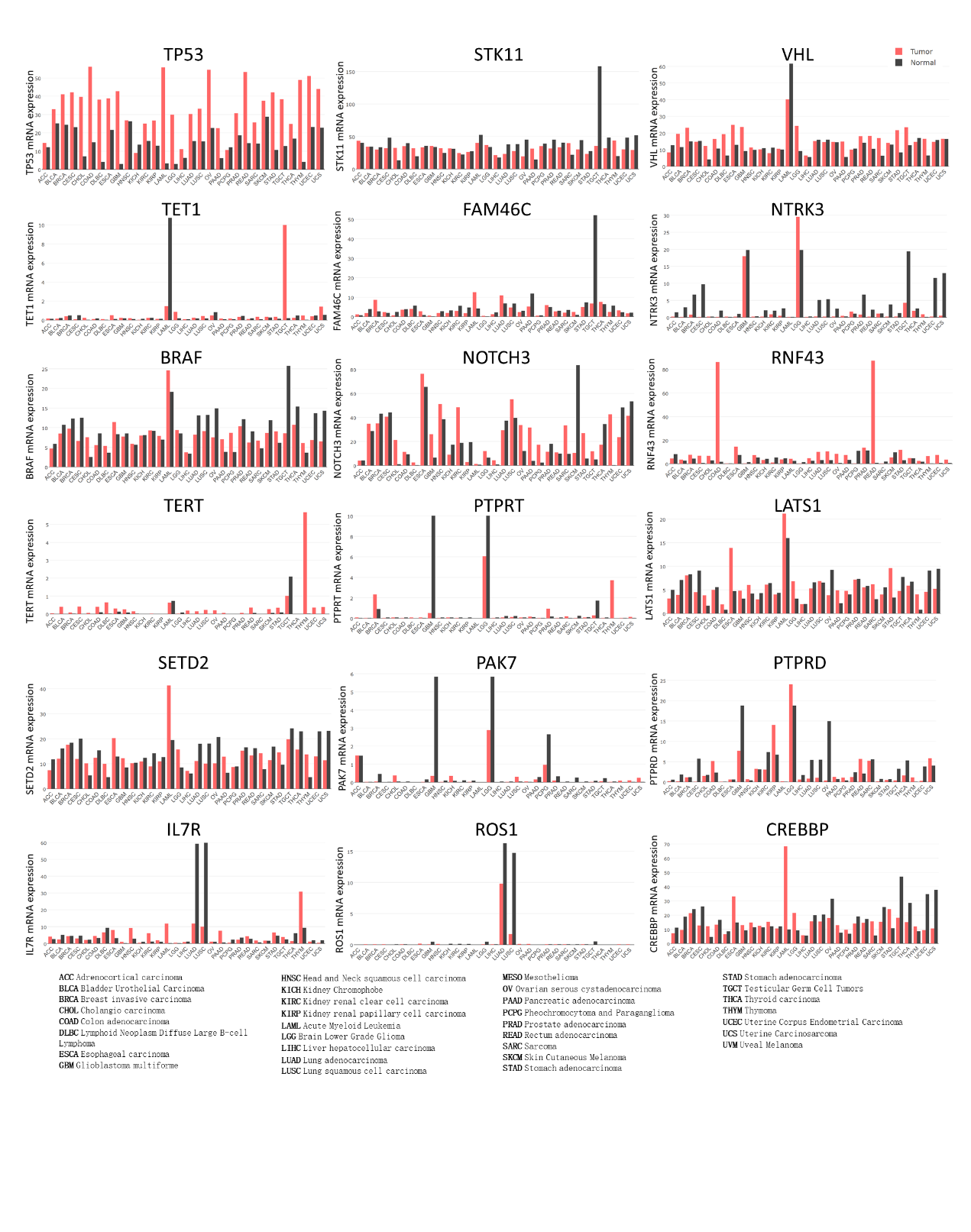


**Figure S5.** The median gene expression profile of the 18 genes in our study in tumor samples and paired normal tissues by GEPIA (Bar plot).


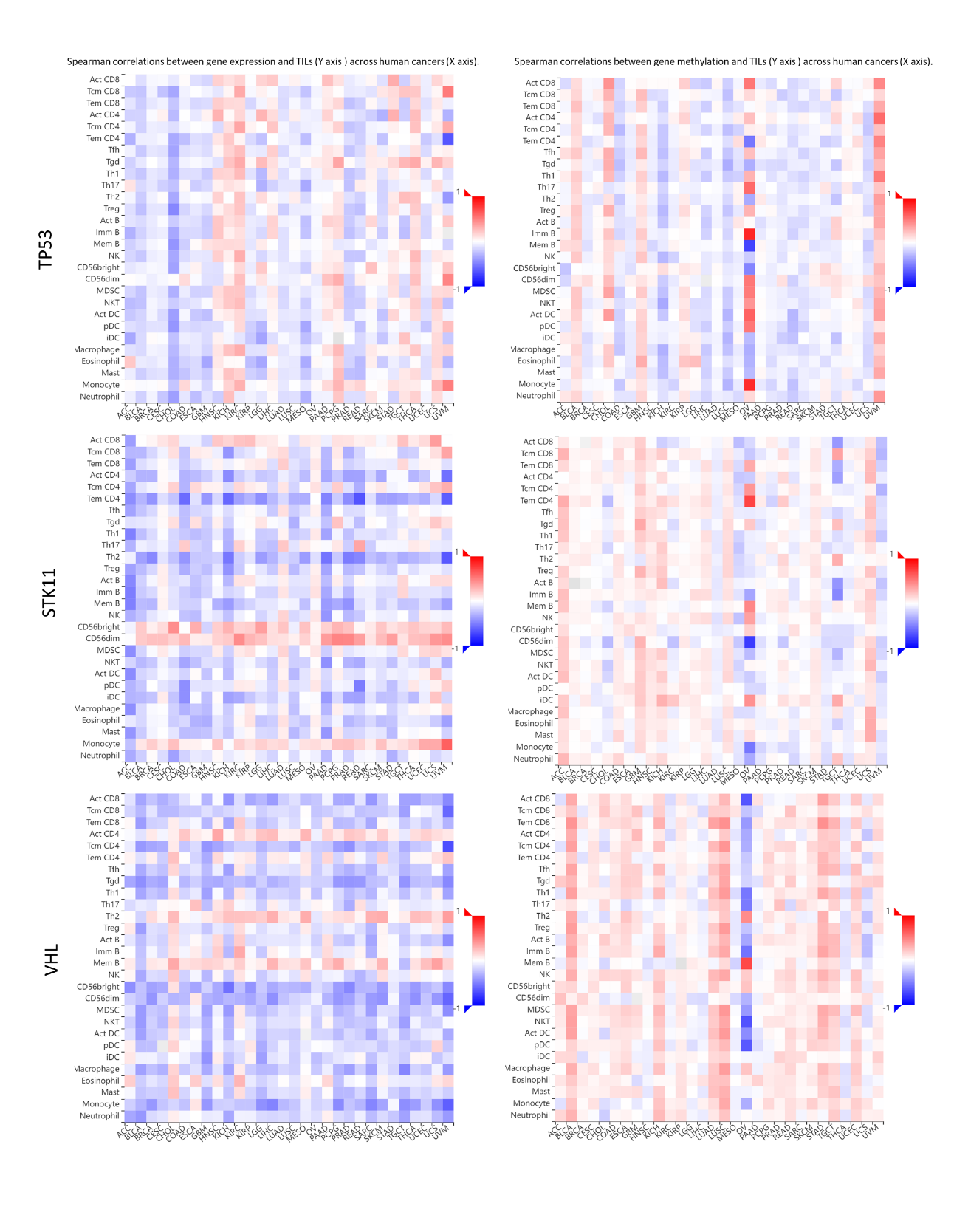


**Figure S6.** Spearman correlations between gene (TP53, STK11, and VHL) methylation and TILs across different cancers.
